# Transcript diversity reflects deleterious RNA processing errors shaped by population size in metazoans

**DOI:** 10.1371/journal.pbio.3003671

**Published:** 2026-03-19

**Authors:** Kai Mi, Lili Guan, Bandhan Sarker, Siliang Song, Tianjiao Zhou, Hongliang Yi, Jianzhi Zhang, Chuan Xu

**Affiliations:** 1 Bio-X Institutes, Key Laboratory for the Genetics of Developmental and Neuropsychiatric Disorders, Ministry of Education, Shanghai Jiao Tong University, Shanghai, China; 2 Department of Otorhinolaryngology Head and Neck Surgery, Shanghai Sixth People’s Hospital Affiliated to Shanghai Jiao Tong University School of Medicine, Shanghai, China; 3 Department of Ecology and Evolutionary Biology, University of Michigan, Ann Arbor, Michigan, United States of America; 4 Key Laboratory of Biodiversity and Environment on the Qinghai-Tibet Plateau, Ministry of Education, Lhasa, China; University of Bath, UNITED KINGDOM OF GREAT BRITAIN AND NORTHERN IRELAND

## Abstract

In eukaryotes, alternative transcription initiation (ATI), alternative splicing (AS), and alternative polyadenylation (APA) result in multiple different transcripts per gene, but the biological significance of the transcript diversity produced remains controversial. Some suggested that this diversity is adaptive, while others contended that it is largely deleterious and arises from molecular errors in transcription and RNA processing. The error hypothesis makes a distinct prediction that is not expected under the adaptive hypothesis: transcript diversity declines with the effective population size (*N*_e_) of the species because natural selection minimizing errors is more effective under larger *N*_e_. By analyzing 166 transcriptomes from 75 metazoans, we report that transcript diversity measured by the percentage uses of minor ATI, AS, and APA sites decreases with *N*_e_ or its proxies. This observation supports the error hypothesis and suggests that metazoan transcript diversity is largely deleterious.

## Abstract 2

Alternative transcription initiation, splicing and polyadenylation generate extensive transcript diversity in eukaryotes, but its evolutionary significance has been disputed. This study analyses 166 transcriptomes across 75 metazoan species to show that transcript diversity generally decreases with effective population size, supporting the view that most transcript diversity reflects deleterious RNA processing errors rather than adaptive functions.

## Introduction

In eukaryotes, alternative transcription initiation (ATI) [1] and alternative polyadenylation (APA) [2] can respectively vary the beginning and end of a transcript produced from a gene, while alternative splicing (AS) can generate different RNA isoforms by selective inclusion or exclusion of exons in mRNA processing [3]. As a result, multiple different RNA transcripts (isoforms) are often produced from a single eukaryotic gene [4,5], generating transcript diversity. These transcripts may vary in their coding sequence, untranslated regions, and/or other regulatory elements [1–3]. ATI, APA, and AS are common phenomena in various eukaryotes such as fungi [6–8], plants [9–11], and animals [12–14]. For example, in humans, > 70% of genes exhibit APA [13], > 50% of genes display ATI [15], and >95% of multi-exon genes show AS [16], resulting in >170,000 transcripts recorded for ~20,000 human protein-coding genes (ENSEMBL genome reference consortium human build 38; GRCh38). ATI, APA, and AS may vary among tissues [17–19], across developmental stages [19–21], and during cell differentiation [22–24], and can contribute to disease [1,25,26].

Despite the universality of ATI, APA, and AS in eukaryotes, the biological significance of the created transcript diversity is debated. Some case studies suggested that different RNA isoforms are functionally distinct. For instance, the human *Lef1* gene [27], mouse *Ighm* gene [28], and Drosophila *Sxl*, *Tra*, and *Dsx* genes [29] have functionally distinct RNA isoforms (and corresponding protein isoforms) produced by ATI, APA, and AS, respectively. Such examples led to the hypothesis that transcript/proteome diversity is generally adaptive and that ATI, APA, and AS are widely used, regulated mechanisms to expand transcript/proteome diversity [3,4,30,31]. However, a competing hypothesis known as the error hypothesis has also been suggested [32–38]. The error hypothesis contends that transcription and RNA processing are error-prone; consequently, the vast majority of the observed transcript diversity reflects molecular errors that not only lower the number of functional molecules and waste energy but may also create cytotoxicity [39]. Several lines of evidence support the error hypothesis [32–36,39,40]. For example, the error hypothesis predicts that transcript diversity is lower in relatively highly expressed genes than in relatively lowly expressed genes because of stronger selection minimizing error rates acting on highly than lowly expressed genes [39]. Empirical data indeed support this prediction [39].

Nonetheless, the level of transcript diversity has not been extensively compared across species. Such a comparison is useful for differentiating between the adaptive and error hypotheses, because the two hypotheses make distinct predictions about the relationship between the level of transcript diversity in a species and the effective population size (*N*_e_) of the species. Specifically, under the error hypothesis, transcript diversity is due to deleterious molecular error, so is disfavored and lowered by natural selection. Because the efficacy of natural selection increases with *N*_e_, we expect transcript diversity to decline with *N*_e_ [41,42]. Under the adaptive hypothesis, however, transcript diversity is beneficial so may be selectively elevated. Under this scenario, transcript diversity is expected to increase with *N*_e_. However, one could also argue that, under the adaptive hypothesis, the optimal level of transcript diversity in a species depends on the specific condition and environment of the species; as a result, no prediction can be made regarding the relationship between the level of transcript diversity and *N*_e_. At any rate, a negative correlation between *N*_e_ and transcript diversity is predicted by the error hypothesis but is not expected under the adaptive hypothesis. Indeed, this prediction was validated by a comparative analysis of AS across 53 species [36].

In the present work, by quantifying ATI, APA, and AS in 166 transcriptomes, we compared levels of transcript diversity among 75 metazoan species spanning a wide range of *N*_e_. We found that transcript diversity generally decreases with *N*_e_ or its proxies, strengthening the previous support of the error hypothesis for AS [36] and providing new evidence for this hypothesis for ATI and APA.

## Results

### Genomic and transcriptomic data

**Fig 1:**
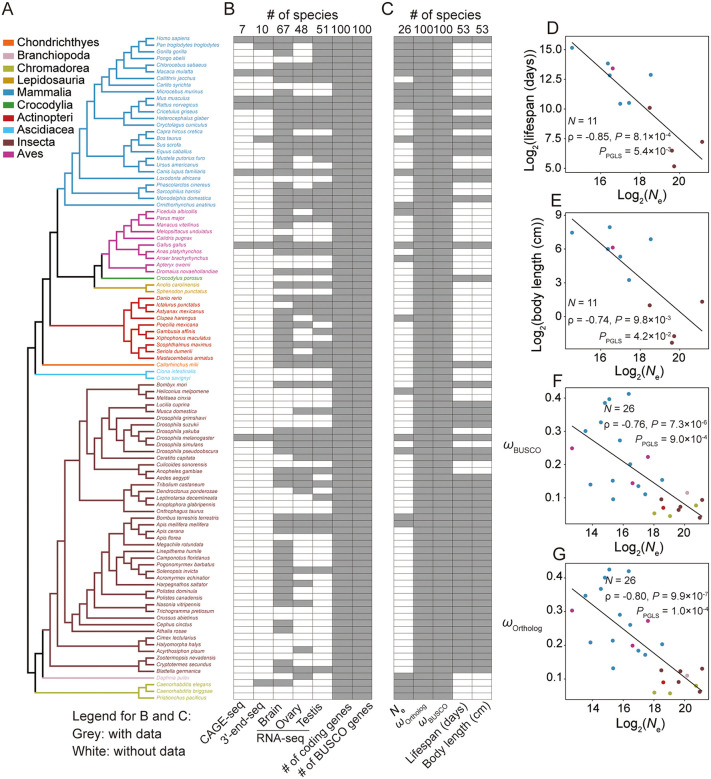
Species phylogeny, genomic and transcriptomic data, and *N*_e_ proxies used in the present study. **(A)** Phylogenetic tree of the 100 metazoan species considered. **(B)** Available genome and transcriptome datasets of each species concerned, including CAGE-seq, 3′-end-seq, RNA-seq, coding genes, and BUSCO genes. **(C)** *N*_e_ and proxies. Spearman’s correlation and Phylogenetic Generalized Least Squares (PGLS) regression between *N*_e_ and life span **(D)**, body length **(E)**, and the nonsynonymous to synonymous substitution rate ratio *ω* computed using all BUSCO genes **(F)** and all one-to-one orthologous genes **(G)** across species. Each dot represents a species, colored according to its clade in **(A)**. The data underlying this Figure can be found in https://doi.org/10.5281/zenodo.18514977.

Based on two recent studies [36,43], we assembled a list of 100 diverse metazoan species with available genome sequences (Fig 1A). We collected publicly available genome, genomic annotation, and transcriptome data from these 100 species (Fig 1B and S1 Data). The transcriptomic data are the basis of transcript diversity estimation and they comprise Cap Analysis of Gene Expression Sequencing (CAGE-seq) data from seven species for direct measurement of ATI, 3′-end-seq data from 10 species for direct measurement of APA, and RNA-seq data from 75 species for detection of AS and prediction of ATI and APA (Fig 1B and S1 Data).

Because transcript diversity estimated from different tissues may not be comparable across species, we focused on three tissues (brain, ovary, and testis) best represented in the transcriptomic data, respectively covering 67, 51, and 48 species (Fig 1B and S1 Data). In interspecific comparisons, we initially analyzed all protein-coding genes in each species (Fig 1 and S1 Data). However, gene set variations among species could introduce a confounder in our comparison. We therefore focused on Benchmarking Universal Single-Copy Orthologs (BUSCO) genes in an additional comparison (Fig 1B and S1 Data), as was done in previous studies [36,43].

### *N*_e_ and proxies

Of the 100 species, 26 have published *N*_e_ (Fig 1C and S2 Data). Given that most species lack published *N*_e_, we resorted to three other parameters as *N*_e_ proxies: the body length, life span, and nonsynonymous to synonymous substitution rate ratio (*ω*). Because larger animals and longer-lived animals tend to have smaller *N*_e_, body length and life span have been used as *N*_e_ proxies [36,44,45]. Under the nearly neutral theory and the neutral assumption of synonymous mutations, *ω* is expected to decline with *N*_e_ [41] so can also be a proxy for *N*_e_. However, when synonymous mutations are frequently non-neutral as has been documented in some species [46], the validity of the above expectation is uncertain; we therefore examined it empirically (see below). We collected body length and life span data from previous studies [36,47] and estimated *ω* for each species (see Materials and methods and S3 Data). We correlated the three proxies with *N*_e_ and confirmed their negative correlations (Fig 1D–1G), as reported previously [36]. Therefore, the error hypothesis predicts that transcript diversity should decline with *N*_e_ or increase with the three *N*_e_ proxies considered here. Note that throughout this study, we used two types of correlation analysis. The first is the simple rank correlation, whereas the second is Phylogenetic Generalized Least Squares (PGLS) regression, which controls for the phylogenetic relationships of the species considered in the data (see Materials and methods).

### Interspecific variation in transcript diversity caused by APA

**Fig 2:**
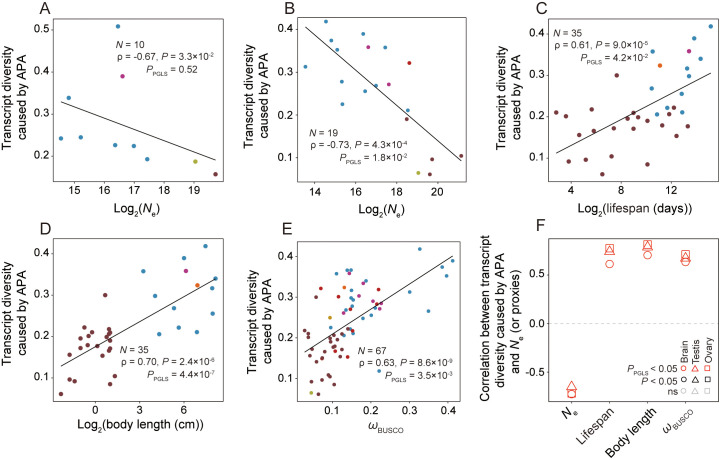
Transcript diversity caused by APA (measured by the mean total percentage usage of minor APA sites per gene) declines with *N*_e_ across species. **(A)** Relationship between *N*_e_ and transcript diversity caused by APA estimated from 3′-end-seq. **(B–E)** Relationship between transcript diversity caused by APA estimated from RNA-seq and *N*_e_ **(B)**, life span **(C)**, body length **(D)**, or *ω* (E) in the brain. *P*-values from Spearman’s correlation and PGLS are shown. *N* represents the number of species included. **(F)** Correlation between transcript diversity caused by APA and *N*_e_ (or proxies) in three tissues. BUSCO genes are used in all panels. The data underlying this Figure can be found in https://doi.org/10.5281/zenodo.18514977.

Accurate detection of APA typically relies on 3′-end RNA sequencing (i.e., 3′-end-seq), which can identify precise APA sites and their relative usages [48]. Several library preparation methods are available for 3′-end-seq [49], such as 3′READS [50], 3P-seq [51], and PAS-seq [52]. However, only a limited number of species have 3′-end-seq data [53]. We collected 3′-end-seq datasets from 10 species with *N*_e_ (S1 Data). For a given gene, let us refer to the most frequently used APA site as its major APA site, which is likely to be functionally the best APA site, and all other APA sites as its minor APA sites. We measured the transcript diversity of the gene caused by APA by the total percentage usage of its minor APA sites, which equals the total 3′-end reads of its minor APA sites divided by the total APA reads of the gene. We then averaged the transcript diversity across all genes considered in a species to represent the overall transcript diversity due to APA for the species. We found a negative correlation between transcript diversity and *N*_e_ across species, regardless of whether we considered all protein-coding genes (S1A Fig) or only BUSCO genes (Fig 2A). However, the negative correlations (or those from PGLS) were not significant, potentially due to the limited number of species in the analyses.

To increase the number of species in the APA analysis, we predicted APA sites using RNA-seq data [48,54]. In particular, TAPAS leverages the Pruned Exact Linear Time algorithm, RNA-seq data, and gene structure information to predict APA sites and their abundances [55] and is known to outperform other tools [56]. We validated the performance of TAPAS by comparing the APA sites identified by 3′-end-seq with those predicted by TAPAS from RNA-seq using a dataset in which CD4 T cells were sequenced by both 3′-end-seq and RNA-seq [57]. We quantified APA site usage levels in both data types (see Materials and methods) and found a significant positive correlation between them (*ρ* > 0.27, *P* < 0.01; S1B Fig). We also computed the total percentage usage of minor APA sites for each gene in both data types and again observed a significant positive correlation between them (*ρ* > 0.16, *P* < 0.01; S1C Fig). To validate the applicability of APA site prediction by TAPAS in multiple species, we acquired RNA-seq datasets from the 10 species with 3′-end-seq data; while 3′-end-seq and RNA-seq were not generated from the same samples, they were from the same tissues in each of these species (S1 Data). We found that the average total percentage usage of minor APA sites per gene is significantly correlated between 3′-end-seq and RNA-seq data across species (*ρ* = 0.79, *P* = 9.8 × 10⁻³; S1D Fig). These results confirm the reliability of predicting APA sites from RNA-seq data.

We previously reported a negative correlation between the total percentage usage of minor APA sites of a gene and the gene expression level in each of five mammals studied, supporting the error hypothesis [32]. To assess whether this pattern extends beyond mammals, we repeated the analysis using APA sites predicted from RNA-seq data and found that the negative correlation persists in 163 of the 166 samples analyzed (S1E Fig), suggesting that this pattern is generally true across animals.

Next, we used RNA-seq data to estimate the average total percentage usage of minor APA sites per BUSCO gene in each of 75 species. We started with the brain because the number of species with RNA-seq data from this tissue is the highest. Across species, the above estimate of species transcript diversity reduces with *N*_e_ (*ρ* = −0.73, *P* = 4.3 × 10^−4^; *P*_PGLS_ = 1.8 × 10^−2^; Fig 2B), but increases with life span (*ρ* = 0.61, *P* = 9 × 10^−5^; *P*_PGLS_ = 4.2 × 10^−2^; Fig 2C), body length (*ρ* = 0.70, *P* = 2.4 × 10^−6^; *P*_PGLS_ = 4.4 × 10^−7^; Fig 2D), and *ω* (*ρ* = 0.63, *P* = 8.6 × 10^−9^; *P*_PGLS_ = 3.5 × 10^−3^; Fig 2E). Similar results were observed for the ovary and testis (Fig 2F). When the above transcript diversity in a species was calculated using all protein-coding genes, the patterns remain qualitatively unchanged (S1F Fig). Hence, patterns of interspecific variation in transcript diversity caused by APA support the error hypothesis.

### Interspecific variation in transcript diversity caused by ATI

Although ATI is ideally assessed by CAGE-seq data [58], such data are available for only several species in our collection (S1 Data). Because ATI can be inferred from RNA-seq data [59,60], we chose to use RNA-seq to compare ATI across species to allow the inclusion of a broader range of species in our analysis. We used SEASTAR, a method known to outperform other tools [60], to predict ATI sites. To validate the SEASTAR prediction, we collected a set of samples sequenced by both CAGE-seq and RNA-seq [61] and, respectively, identified ATI sites from CAGE-seq and from RNA-seq using SEASTAR for all protein-coding genes. Analogous to the APA analysis, we quantified the expression level for each ATI site, the total percentage usage of minor ATI sites for each gene, and the average total percentage usage of minor ATI sites per gene in a species (see Materials and methods), and found them to respectively exhibit a significant, positive correlation between estimates from CAGE-seq and those from RNA-seq (*ρ* > 0.32, *P* ≤ 0.02; S2A–S2C Fig). These findings support the reliability of predicting ATI site usage from RNA-seq data.

Our previous study in humans and mice showed that the total percentage usage of minor ATI sites of a gene declines with the gene expression level, supporting the error hypothesis of ATI [33]. In the three tissues of every species investigated in the present study, the above trend is observed (S2D Fig).

**Fig 3:**
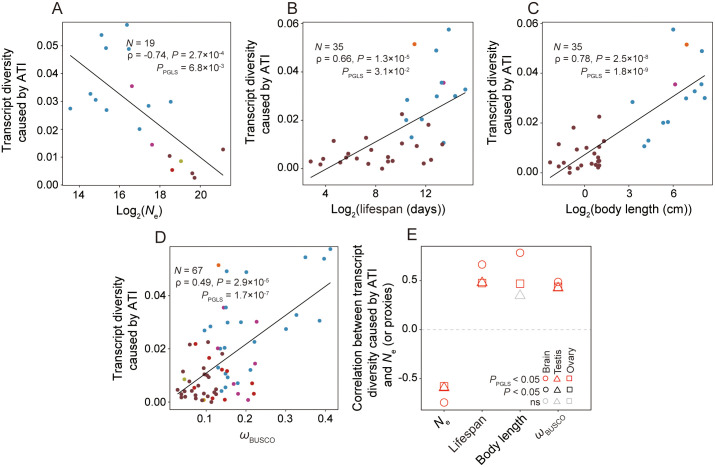
Transcript diversity caused by ATI (measured by the mean total percentage usage of minor ATI sites per gene from RNA-seq) declines with *N*_e_ across species. **(A–D)** Relationship between transcript diversity caused by APA and *N*_e_ **(A)**, life span **(B)**, body length **(C)**, or *ω* **(D)** in the brain. *P*-values from Spearman’s correlation and PGLS are shown. *N* represents the number of species included. **(E)** Correlation between transcript diversity caused by ATI and *N*_e_ (or proxies) in three tissues. BUSCO genes are used in all panels. The data underlying this Figure can be found in https://doi.org/10.5281/zenodo.18514977.

Next, we calculated the average total percentage usage of minor ATI sites per gene for the BUSCO genes of a species using RNA-seq data and correlated it with *N*_e_ across species. Indeed, a significant, negative correlation was observed across 19 species (*ρ* = −0.74, *P* = 2.7 × 10^−4^; *P*_PGLS_ = 6.8 × 10^−3^; Fig 3A). We similarly observed a positive correlation when *N*_e_ is replaced with life span (*ρ* = 0.66, *P* = 1.3 × 10^−5^; *P*_PGLS_ = 3.1 × 10^−2^; Fig 3B), body length (*ρ* = 0.78, *P* = 2.5 × 10^−8^; *P*_PGLS_ = 1.8 × 10^−9^; Fig 3C), or *ω* (*ρ* = 0.49, *P* = 2.9 × 10^−5^; *P*_PGLS_ = 1.7 × 10^−7^; Fig 3D). Similar results were obtained for the testis and ovary for BUSCO genes (Fig 2E) or all protein-coding genes (S2E Fig).

### Interspecific variation in transcript diversity caused by AS

Previous studies have shown that AS is noisy [62] and mostly nonadaptive [34], consistent with the error hypothesis. To compare transcript diversity caused by AS across species, we assembled the transcripts for a gene and quantified the expression level of each transcript of the gene using StringTie, a widely-used tool outperforming others in the accuracy of both assembly and expression level measurement [63]. We then computed for each gene its transcript diversity due to AS by dividing the total splicing amount of all minor RNA splicing isoforms by the total splicing amount of all RNA splicing isoforms of the gene. Here, the splicing amount of an RNA splicing isoform is the total number of reads covering all splicing junctions of the isoform.

The error hypothesis predicts that the transcript diversity of a gene caused by AS should decrease with the expression level of the gene, because more highly expressed genes are subject to stronger selection against splicing error [64]. Indeed, we observed negative correlations in 158 of 166 samples (S3A Fig). These results confirm the previous findings from a limited number of species [34] and suggest that the error hypothesis of AS is broadly supported in animals.

**Fig 4:**
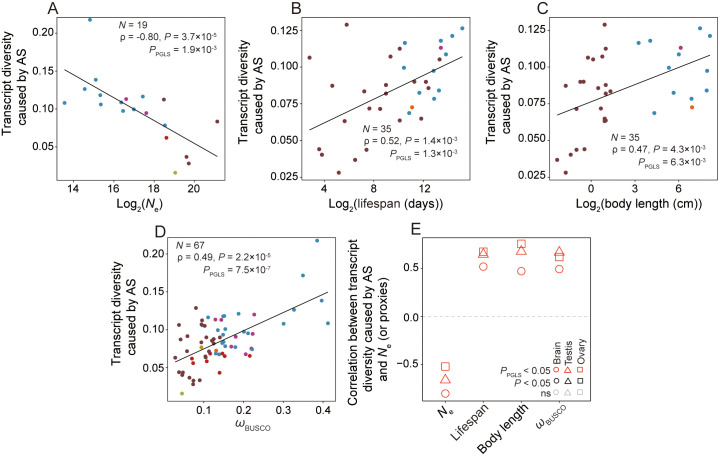
Transcript diversity caused by AS (measured by the mean total percentage usage of splicing junctions in minor splicing isoforms per gene) declines with *N*_e_ across species. **(A–D)** Relationship between transcript diversity caused by AS and *N*_e_ **(A)**, life span **(B)**, body length **(C)**, or *ω* **(D)** in the brain. *P*-values from Spearman’s correlation and PGLS are shown. *N* represents the number of species included. **(E)** Correlation between transcript diversity caused by AS and *N*_e_ (or proxies) in three tissues. BUSCO genes are used in all panels. The data underlying this Figure can be found in https://doi.org/10.5281/zenodo.18514977.

Next, we calculated the mean transcript diversity (caused by AS) per gene for each species among BUSCO genes using data from the brain tissue. We found that this quantity significantly decreases with *N*_e_ (Fig 4A), but increases with life span (Fig 4B), body length (Fig 4C), and *ω* (Fig 4D). Similar results were observed for the ovary and testis (Fig 4E). These patterns remain qualitatively unchanged (S3B Fig) when all protein-coding genes were analyzed. Thus, consistent with a previous study [36], our across-species comparison of AS supports the error hypothesis.

## Discussion

Transcript diversity primarily arises from ATI, APA, and AS. Although past studies have provided substantial genomic evidence for the error hypothesis of ATI [33], APA [32,65], and AS [34], these studies focused on a small number of species. As a result of this limitation and an increasing number of reports of cases of functional ATI, APA, or AS, the general biological significance of transcript diversity remains controversial. In the present study, we expanded the analysis to 75 species and showed that the previous finding from a small number of species generally hold across animals. More importantly, we found that the transcript diversity of a species declines with the species’ *N*_e_ or its proxies, as predicted by the error hypothesis.

A central theoretical underpinning of the non-adaptive paradigm relevant to our results is the drift-barrier model, which was first proposed by Lynch [66] to explain the mutation rate variation across species. This model predicts that the efficacy of selection is limited due to genetic drift and mutation bias, such that phenotypic traits of species with smaller *N*_e_, where drift is more potent, are less optimized than those of species with larger *N*_e_ [42]. For transcript diversity, the drift-barrier model predicts that errors in transcriptional and post-transcriptional processing (e.g., incorrect AS, imprecise polyadenylation, or aberrant transcription initiation) that generate non-functional or weakly deleterious transcript isoforms will persist at higher frequencies in species with smaller *N*_e_. Our observation that transcript diversity declines with *N*_e_ (and its proxies) confirms this prediction and hence supports the drift-barrier model.

Comparative studies across species often encounter confounding factors that could bias the outcome. For instance, estimating transcript diversity in this study relies on transcript annotations, which vary in completeness across species, with model or well-studied organisms typically having more comprehensive annotations, potentially introducing an interspecific bias. To minimize this bias, we performed de novo transcript assembly for each species using RNA-seq data, ensuring uniform annotation processes and quality (see Materials and methods). Although our approach may reduce annotation quality for model organisms, it mitigates the potential interspecific bias. Indeed, the observed patterns are unaltered by including (Figs 2E, 3D, and 4D) or excluding (S4 Fig) model species. Similarly, genome size and complexity can influence genome annotations and transcript diversity assessments. To address this issue, we employed BUSCO genes. These genes are single-copy highly conserved orthologs that are unaffected by genome size or complexity across species, ensuring comparability among taxa. Indeed, when using BUSCO genes to compute transcript diversity, we found that our conclusions hold regardless of whether genome size and complexity are controlled or not (S5 Fig). Thus, our results are robust to the above potential confounding factors in multispecies comparisons.

As mentioned, Benitiere and colleagues (2024) also reported a negative correlation between *N*_e_ and transcript diversity caused by AS across 53 species [36]. Nevertheless, our methodology differs from Benitiere and colleagues’s in several aspects. First, we calculated transcript diversity at the gene level, whereas Benitiere and colleagues measured it at the intron level. Second, Benitiere and colleagues combined multiple RNA-seq datasets to detect splicing events, which improved splicing event detection but introduced heterogeneity among tissues. By contrast, we compared the same tissue across species, which made the interspecific comparison fairer. Third, Benitiere and colleagues analyzed 53 species, most being insects, while our analysis encompassed 75 animals with a broader phylogenetic sampling. Fourth, in addition to the three *N*_e_ proxies used by Benitiere and colleagues in the correlation analysis, our study also used *N*_e_ from 26 species. Notwithstanding these methodological differences, the findings of the two studies are consistent.

In the debate about the biological significance of AS, several authors noted a significant positive correlation between the amount of AS of a species and its organismal complexity measured by the number of cell types [67,68]. Chen and colleagues [68] reported that this correlation remains even after the control for the species’ *N*_e_, suggesting that AS is adaptive and is at least partially responsible for organismal complexity. Their analysis was recently criticized by Benitiere and colleagues [36] for using nucleotide diversity at synonymous sites (*π*_S_) as a proxy for *N*_e_. Synonymous mutations are often non-neutral [46], but even when they are neutral, *π*_S_ is determined by both *N*_e_ and the mutation rate per site per generation, the latter of which varies across species [42]. Hence, *π*_S_ is not an appropriate proxy for *N*_e_. While Benitiere and colleagues suggested that *ω* would be a more appropriate proxy for *N*_e_, they did not perform the actual partial correlation analysis. We therefore investigated the relationships among transcript diversity caused by AS, number of cell types, and *ω* across 12 species for which all three estimates are available in our brain tissue dataset (S4 Data). Consistent with the finding of Chen and colleagues [68], we observed a positive correlation between the transcript diversity caused by AS and the number of cell types across species even after the control for *ω*, but this partial correlation did not reach statistical significance in the PGLS analysis (S6 Fig). That is, after the control for the phylogenetic relationships in the data and *ω* (as a proxy for *N*_e_), there is no significant partial correlation between organismal complexity measured by the number of cell types and transcript diversity caused by AS. We note that, even if the above partial correlation is significant, it does not mean that AS underlies organismal complexity. This is because, to demonstrate that AS contributes to organismal complexity, one needs to show that AS varies among cell types and plays a role in the functional diversity among cell types, which will be an interesting direction to pursue in the future when AS can be reliably assessed from single-cell RNA-seq data.

It is important to note that beyond APA, ATI, and AS, there are other variations in gene expression that result in gene product diversity, including post-transcriptional modifications (e.g., RNA editing [69] and m^5^C modifications [37]), translation variations (e.g., alternative translation initiation [70], mistranslation [71], and stop-codon read-through [72]), and post-translational modifications (e.g., phosphorylation). Notably, a recent study of the mis-transcription rate reveals a narrow range of variation across the tree of life [73]. Hence, it would be highly valuable to conduct cross-species comparisons as performed here for additional types of transcript diversity when appropriate data become available from a sufficient number of species.

## Materials and methods

### Genomes, transcriptomes, and *N*_e_ estimates

The species used in this study were sourced from Zhang and colleagues and Bénitière and colleagues [36,43]. The *N*_e_ estimates were acquired from the references listed in S2 Data, where *N*_e_ for the majority of species was inferred from measures of presumably neutral polymorphism (*π*) and germline mutation rate (*µ*), under the assumption of mutation-drift equilibrium, following the relationship *N*_e_ = *π*/(4*µ*) for diploid species. Data of body length, life span, and number of cell types were obtained from previous studies [36,47]. Reference genomes, protein sequences, cDNA sequences, and RNA-seq data were downloaded from the ENSEMBL (release 100) [74] and NCBI [75]. Accession numbers of RNA-seq samples are listed in S1 Data.

### Phylogenetic tree

We obtained the phylogenetic tree of the species concerned from the Open Tree of Life [76]. We used BUSCO to identify single-copy protein-coding orthologous genes conserved in all 100 species, concatenated the protein sequences of the single-copy orthologs in each species, and performed multiple sequence alignments using MUSCLE [77] with the default parameters. Poorly aligned regions in the resulting alignment were removed using trimAl [78] with the “-automated1” method. The branch lengths of the tree were calculated using codeml from PAML with the JTT substitution model (“seqtype = 2, runmode = 0, model = 2, aaRateFile = jones.dat”).

### *ω* estimation

For each focal species, we selected a triplet consisting of three species to estimate its *ω* value. Specifically, the triplet included the focal species, a closely related ingroup species, and an outgroup species. For instance, the *ω* values of human and chimpanzee were estimated using the triplet ((human, chimpanzee), gorilla), whereas the *ω* value for gorilla was estimated using ((human, gorilla), orangutan). For each triplet, we identified 1:1:1 orthologous protein-coding genes among the three species using OrthoFinder [79]. These orthologous genes were concatenated in the same order to construct a supergene. Multiple sequence alignments of the supergene protein sequences were performed using MUSCLE [77] with default parameters, and codon alignments were generated using TranAlign from the EMBOSS package [80]. Finally, we estimated the *ω* value of the supergene using the branch model (model = 1) implemented in codeml from the PAML package [81]. The resulting *ω* value of the supergene was taken as the *ω* value for that species. When focusing on BUSCO genes, we used only the BUSCO orthologs in estimating *ω*.

### RNA-seq data processing

We utilized fastp [82] to perform quality control of RNA-seq raw reads and then aligned high-quality reads to the corresponding reference genomes using STAR 2.7.10a [83]. Read counts of a gene in each sample were obtained by featureCounts [84] based on the read alignment generated by STAR 2.7.10a. The total exon length of a gene, calculated using a custom script, is defined as the effective length of the gene. Finally, the expression level of a gene is calculated by Transcripts Per Million (TPM) [85]. To mitigate potential biases arising from differences in genome annotation across species, we used StringTie [63] to reassemble gene transcripts from RNA-seq data as reference RNA isoforms.

### APA data processing

3′-end-seq data were downloaded from NCBI (S1 Data). Raw reads were inspected using FastQC and adapters were removed by Cutadapt. Next, clean reads were aligned to the corresponding reference genomes using Bowtie2 [86] with default parameters. Uniquely aligned reads were processed to define APA sites. APA sites supported by fewer than two 3′-end-seq reads were excluded from further analysis, and the remaining sites located within 30 base pairs of one another were merged into a single cluster. The APA site with the highest read count within each cluster represented the cluster, and the total number of 3′-end-seq reads mapped to all APA sites within a cluster was considered the expression abundance of the APA site. An APA site was assigned to a gene if it was mapped within the region spanning from the 5′-end of the gene to 1,000 bp downstream of the 3′-end of the gene. APA sites mapped to multiple genes were excluded from further analysis. The expression level of an APA site was calculated by dividing the total number of reads mapped to the site by the total number of reads from the corresponding library that were successfully mapped to the genome. The TAPAS [55] pipeline with default parameters was used to predict APA sites. For APA sites identified by 3′-end-seq or predicted from RNA-seq, we consider the APA site with the highest expression level in a gene to be the major APA site of the gene, while other APA sites were considered minor APA sites. The total percentage usage of minor APA sties in a gene was calculated by dividing the total expression level of minor APA sites by the total expression level of all APA sites of the gene.

### ATI data processing

CAGE-seq data were downloaded from FANTOM5 [87] and NCBI (S1 Data). Raw CAGE-seq reads were inspected using fastQC and adapters were removed using Cutadapt. rRNAdust was then used to filter out rRNA reads. Next, the cleaned CAGE-seq reads were aligned to the genome using HISAT2 [88] with the default setting. Uniquely aligned reads were processed with the CAGEr [89] package and converted to quantified CAGE transcriptional start site (CTSS) coordinates. CTSSs supported by fewer than two CAGE-seq reads were excluded from further analysis. Remaining CTSSs located within 30 base pairs of one another were merged into a single cluster. The CTSS with the highest read count within each cluster was designated as the representative position of the cluster, referred to as an ATI site. The total number of CAGE-seq reads mapped to all CTSSs within a cluster was defined as the expression abundance of the corresponding ATI site. An ATI site was assigned to a gene if it was mapped within the region spanning from 1,000 bp upstream of the 5′-end of the gene to the 3′-end of the gene. ATI sites mapped to multiple genes were excluded from further analysis. The expression level of an ATI site was calculated by dividing the total number of reads mapped to the site by the total number of reads from the corresponding library that were successfully mapped to the genome. The SEASTAR [60] pipeline with default parameters was used to predict ATI sites from RNA-seq data. ATI sites with the first exon coverage of less than two reads were excluded. The expression level of an ATI site was calculated by dividing its first exon coverage by the product of the length of the exon and the total number of reads mapped to the genome in the library. For ATI sites identified by CAGE-seq or predicted from RNA-seq, the ATI site with the highest expression level for a gene was considered the major ATI site of the gene, while all other ATI sites were considered minor sites. The total percentage usage of minor ATI sties of a gene was computed by dividing the total expression level of minor ATI sites by the total expression level of all ATI sites of the gene.

### AS data processing

The splicing junctions and expression level of each RNA isoform in a gene were respectively assembled and measured using StringTie [63] after STAR alignment. The isoform with the highest TPM in a gene was regarded as the major RNA transcript of the gene, while other isoforms were considered minor RNA transcripts. For junctions that are shared between multiple transcripts, we reassigned junction reads to these transcripts based on their expression levels. The total percentage usage of junctions in minor RNA splicing isoforms was calculated by dividing the splicing amount in minor RNA splicing isoforms of a gene by the total splicing amount of the gene.

### Data analysis

Data analysis was conducted using R statistical software (v4.2). To take phylogenetic inertia into account, we performed PGLS regression [36] by R package “caper” when conducting cross-species correlations.

## Supporting information

S1 Fig Analysis of transcript diversity caused by APA (measured by total percentage usage of minor APA sites) using 3′-end-seq and RNA-seq. **(A)** Correlation between *N*_e_ and transcript diversity caused by APA estimated from 3′-end-seq across seven metazoans. All protein-coding genes are used in the analysis. **(B)** Spearman’s correlation between the expression level of an APA site quantified by 3′-end-seq and that predicted by RNA-seq. **(C)** Spearman’s correlation between the total percentage usage of minor APA sites in a gene quantified by 3′-end-seq and that predicted by RNA-seq. The X-axes in **(B)** and **(C)** show the SRA accession numbers of RNA-seq and 3′-end-seq data from the same sample. **(D)** Spearman’s correlation between transcript diversity caused by APA in a species qualified by 3′-end-seq and that predicted by RNA-seq. **(E)** Spearman’s correlation between the gene expression level and the total percentage usage of minor APA sites across genes in each of three tissues in each of 75 species. Each row represents a species. **(F)** Correlation between transcript diversity caused by APA and *N*_e_, life span, body length, or *ω* across species in three tissues. All protein-coding genes are used in (F). The data underlying this Figure can be found in https://doi.org/10.5281/zenodo.18514977.
(TIF)

S2 Fig Analysis of transcript diversity caused by ATI (measured by the total percentage usage of minor ATI sites) using CAGE-seq and RNA-seq. **(A)** Spearman’s correlation between the expression level of an ATI site quantified by CAGE-seq and that predicted by RNA-seq. **(B)** Spearman’s correlation between the total percentage usage of minor ATI sites in a gene quantified by CAGE-seq and that predicted by RNA-seq. The X-axes in (A) and (B) show the SRA accession numbers of RNA-seq and CAGE-seq data from the same sample. **(C)** Spearman’s correlation between transcript diversity caused by ATI in a species quantified by CAGE-seq and that predicted by RNA-seq. Each dot is a species. **(D)** Spearman’s correlation between the gene expression level and the total percentage usage of minor ATI sites across genes in each of three tissues in each of 75 species. Each row represents a species. **(E)** Correlation between transcript diversity caused by ATI and *N*_e_, life span, body length, or *ω* across species in three tissues. All protein-coding genes are used in (E). The data underlying this Figure can be found in https://doi.org/10.5281/zenodo.18514977.
(TIF)

S3 Fig Analysis of transcript diversity caused by AS (measured by the total percentage usage of splicing junctions in minor RNA splicing isoforms). **(A)** Spearman’s correlation between the gene expression level and the total percentage usage of splicing junctions in minor RNA splicing isoforms across genes in each of three tissues in each of 75 species. Each row represents a species. **(B)** Correlation between transcript diversity caused by ATI and *N*_e_, life span, body length, or ω across species in three tissues. All protein-coding genes are used in (B). The data underlying this Figure can be found in https://doi.org/10.5281/zenodo.18514977.
(TIF)

S4 Fig Correlation between transcript diversity caused by APA (A), ATI (B), and AS (C) and *ω* after removing 9 model species (*Homo sapiens*, *Macaca mulatta*, *Mus musculus*, *Rattus norvegicus*, *Gallus gallus*, *Danio rerio*, *Drosophila melanogaster*, *Aedes aegypti*, and *Caenorhabditis elegans*). The data underlying this Figure can be found in https://doi.org/10.5281/zenodo.18514977.
(TIF)

S5 Fig Partial correlation between transcript diversity caused by APA (A), ATI (B), and AS (C) and *ω* after the control for genome size and complexity. Dots represent the raw data. The data underlying this Figure can be found in https://doi.org/10.5281/zenodo.18514977.
(TIF)

S6 Fig Partial correlation between transcript diversity caused by AS and the number of cell types after the control for *ω.* Dots represent the raw data. The data underlying this Figure can be found in https://doi.org/10.5281/zenodo.18514977.
(TIF)

S1 Data Available datasets of transcriptomes and genomes for the species investigated. (XLSX)

S2 Data *N*_e_ of 26 metazoans reported in previous studies. (DOCX)

S3 Data *N*_e_ and proxies. (XLSX)

S4 Data Twelve species with available information about the number of cell types. (XLSX)

## Abbreviations

APA: alternative polyadenylation
BUSCO: Benchmarking Universal Single-Copy Orthologs
AS: alternative splicing
ATI: alternative transcription initiation
CAGE-seq: Cap Analysis of Gene Expression Sequencing
CTSS: CAGE transcriptional start site
PGLS: Phylogenetic Generalized Least Squares
TPM: Transcripts Per Million
