# Supplementary figures and images for: Transcript diversity reflects deleterious RNA processing errors shaped by population size in metazoans
Source: PLoS Biol. 2026 Mar 19;24(3):e3003671. doi: 10.1371/journal.pbio.3003671 (PMC13001929; doi:10.1371/journal.pbio.3003671)

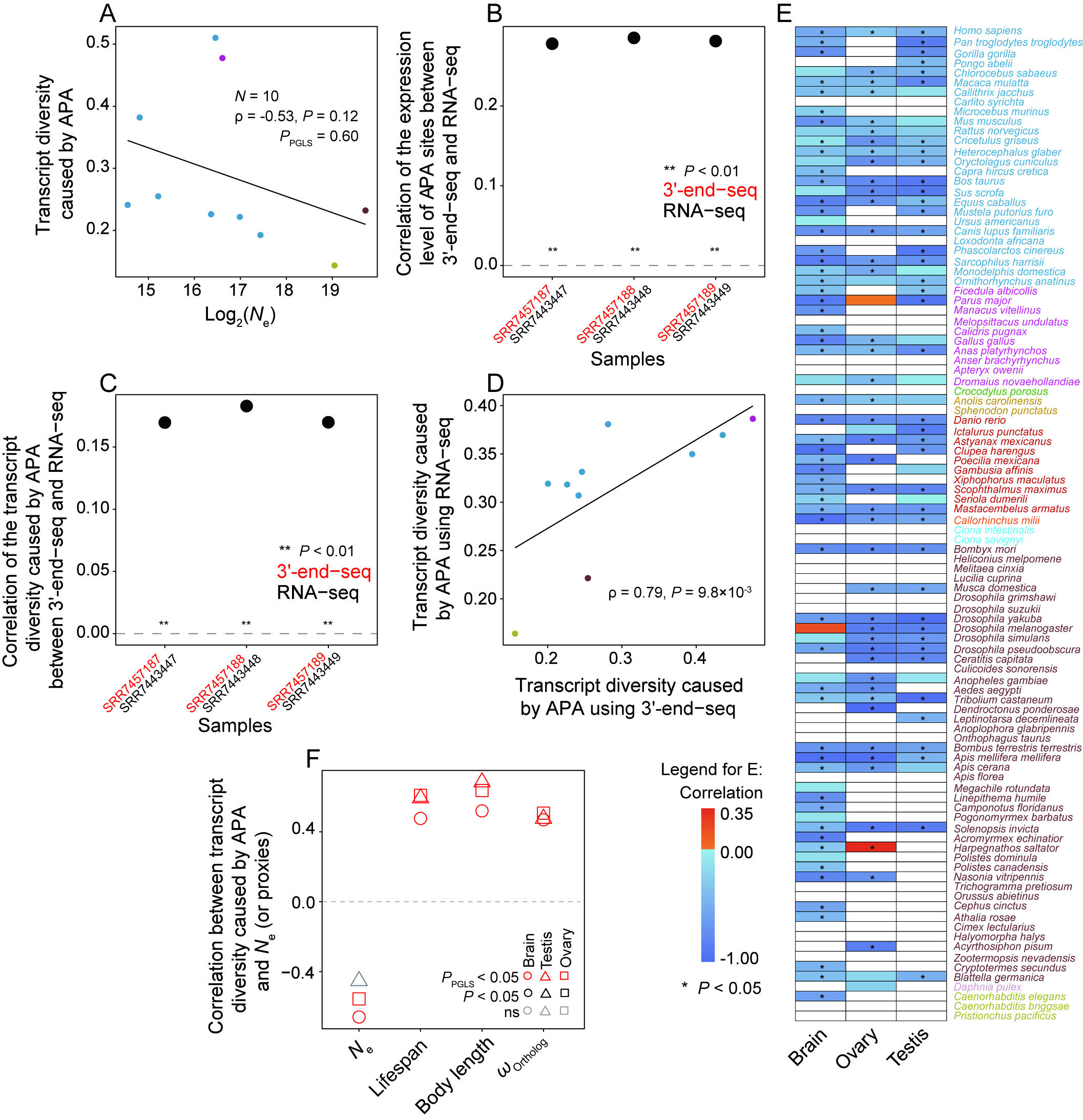

Supplement: S1 Fig — (A) Correlation between Ne and transcript diversity caused by APA estimated from 3′-end-seq across seven metazoans. All protein-coding genes are used in the analysis. (B) Spearman’s correlation between the expression level of an APA site quantified by 3′-end-seq and that predicted by RNA-seq. (C) Spearman’s correlation between the total percentage usage of minor APA sites in a gene quantified by 3′-end-seq and that predicted by RNA-seq. The X-axes in (B) and (C) show the SRA accession numbers of RNA-seq and 3′-end-seq data from the same sample. (D) Spearman’s correlation between transcript diversity caused by APA in a species qualified by 3′-end-seq and that predicted by RNA-seq. (E) Spearman’s correlation between the gene expression level and the total percentage usage of minor APA sites across genes in each of three tissues in each of 75 species. Each row represents a species. (F) Correlation between transcript diversity caused by APA and Ne, life span, body length, or ω across species in three tissues. All protein-coding genes are used in (F). The data underlying this Figure can be found in https://doi.org/10.5281/zenodo.18514977. (TIF) [file pbio.3003671.s001.tif]

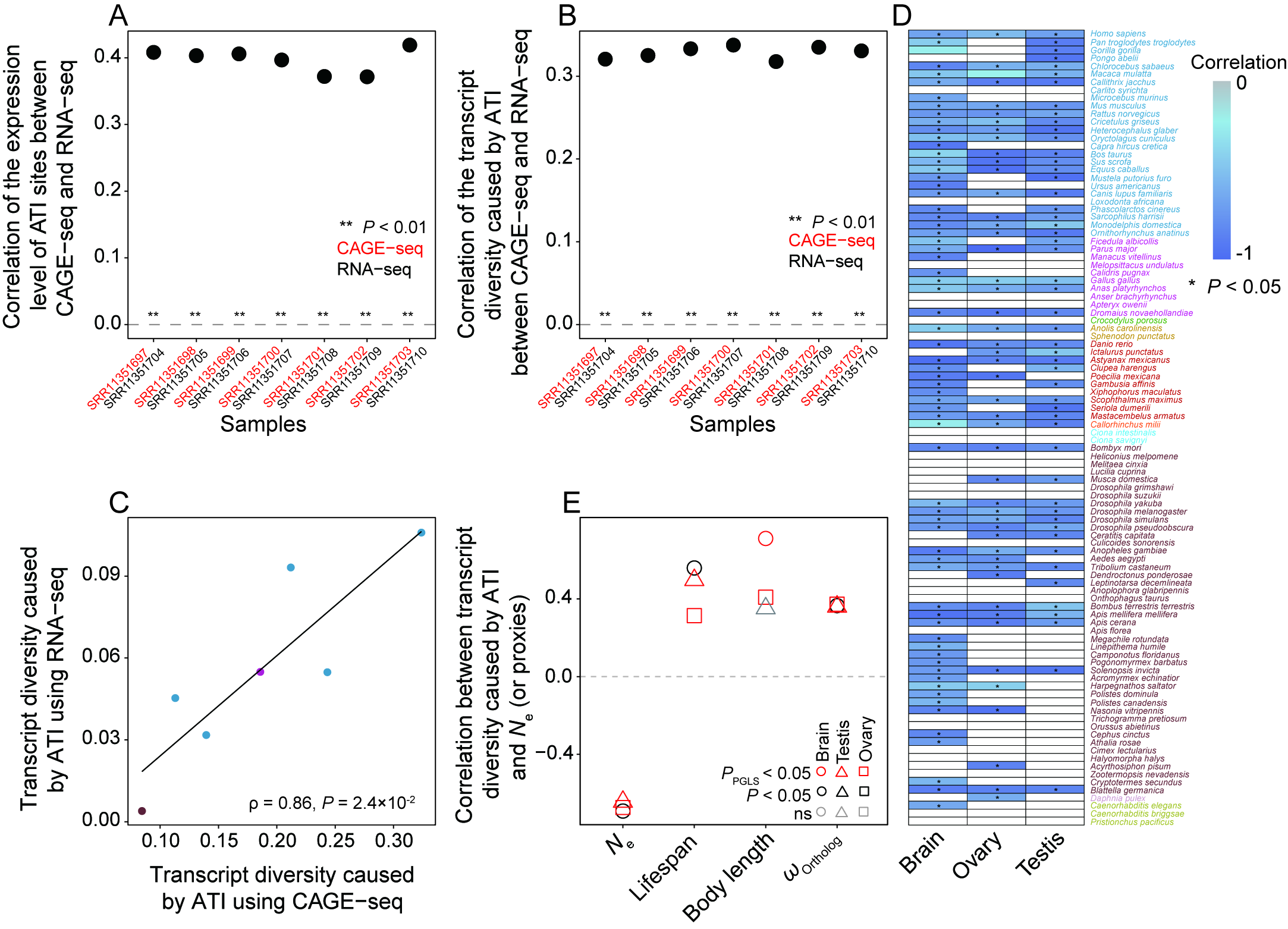

Supplement: S2 Fig — (A) Spearman’s correlation between the expression level of an ATI site quantified by CAGE-seq and that predicted by RNA-seq. (B) Spearman’s correlation between the total percentage usage of minor ATI sites in a gene quantified by CAGE-seq and that predicted by RNA-seq. The X-axes in (A) and (B) show the SRA accession numbers of RNA-seq and CAGE-seq data from the same sample. (C) Spearman’s correlation between transcript diversity caused by ATI in a species quantified by CAGE-seq and that predicted by RNA-seq. Each dot is a species. (D) Spearman’s correlation between the gene expression level and the total percentage usage of minor ATI sites across genes in each of three tissues in each of 75 species. Each row represents a species. (E) Correlation between transcript diversity caused by ATI and Ne, life span, body length, or ω across species in three tissues. All protein-coding genes are used in (E). The data underlying this Figure can be found in https://doi.org/10.5281/zenodo.18514977. (TIF) [file pbio.3003671.s002.tif]

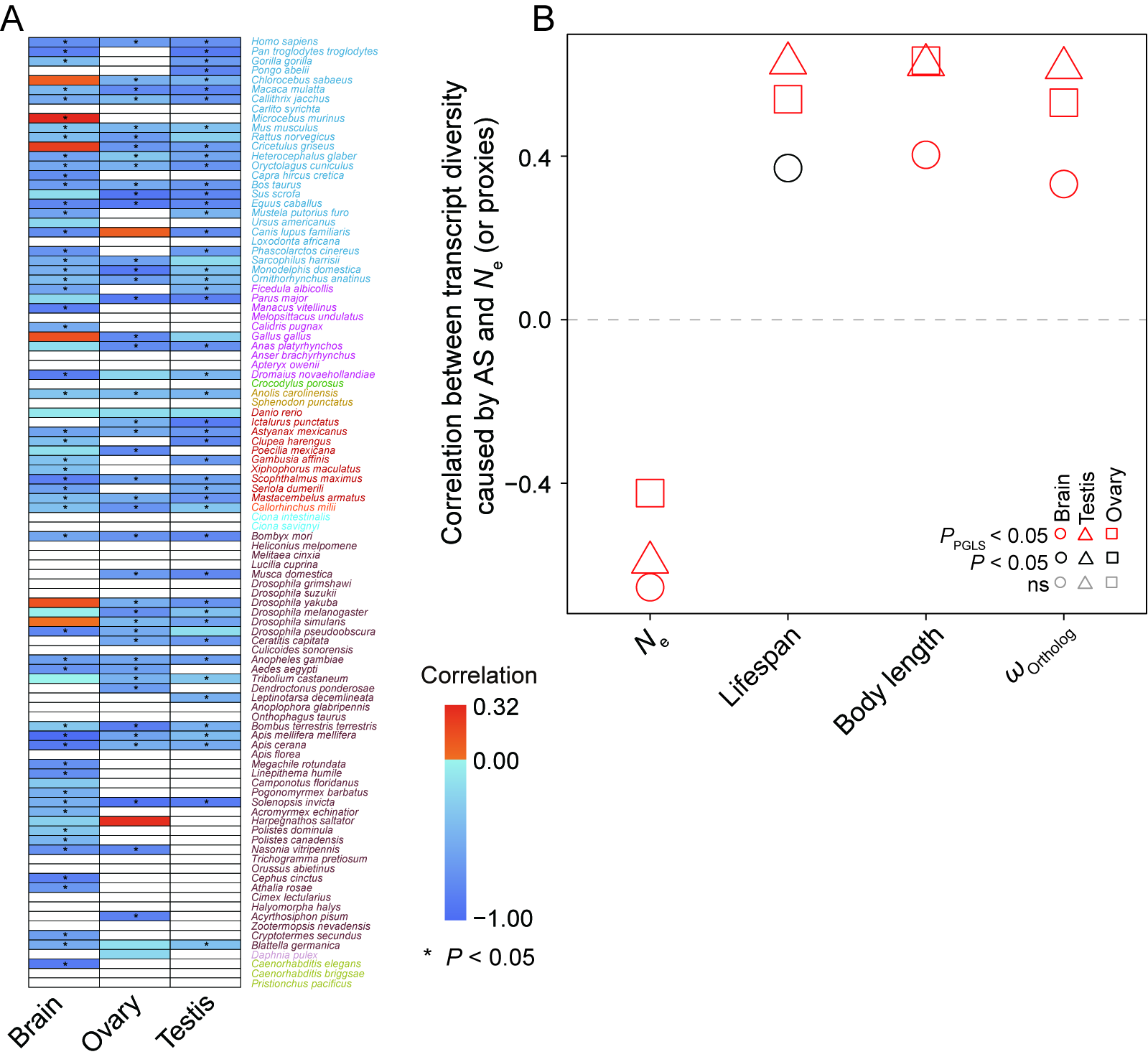

Supplement: S3 Fig — (A) Spearman’s correlation between the gene expression level and the total percentage usage of splicing junctions in minor RNA splicing isoforms across genes in each of three tissues in each of 75 species. Each row represents a species. (B) Correlation between transcript diversity caused by ATI and Ne, life span, body length, or ω across species in three tissues. All protein-coding genes are used in (B). The data underlying this Figure can be found in https://doi.org/10.5281/zenodo.18514977. (TIF) [file pbio.3003671.s003.tif]

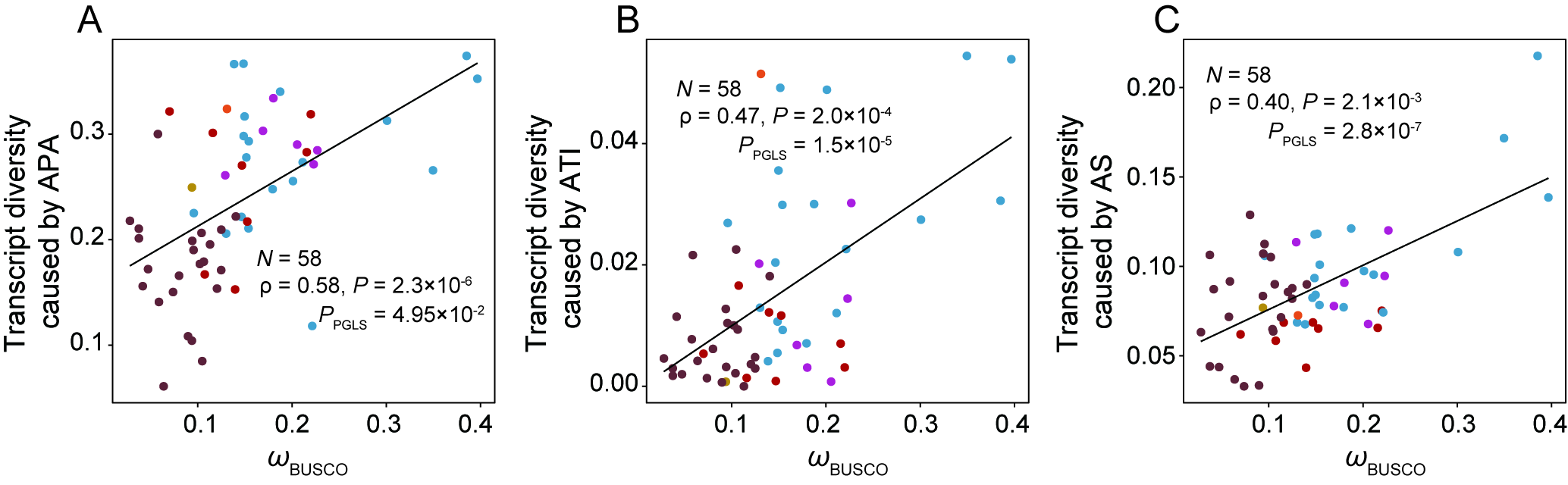

Supplement: S4 Fig — The data underlying this Figure can be found in https://doi.org/10.5281/zenodo.18514977. (TIF) [file pbio.3003671.s004.tif]

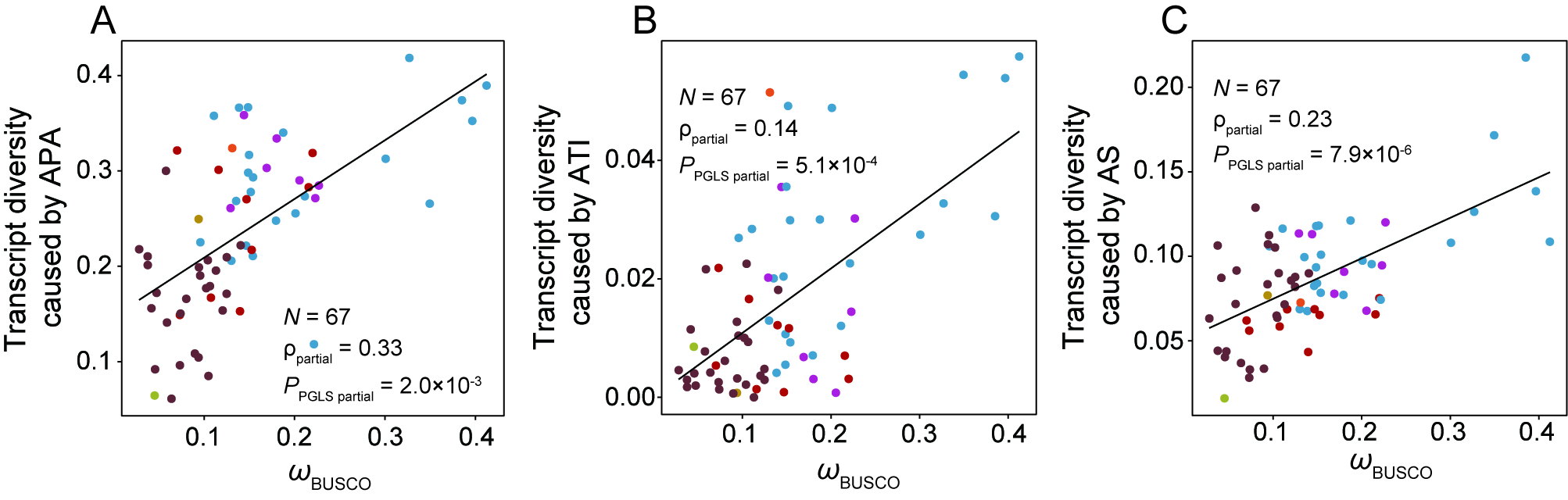

Supplement: S5 Fig — Dots represent the raw data. The data underlying this Figure can be found in https://doi.org/10.5281/zenodo.18514977. (TIF) [file pbio.3003671.s005.tif]

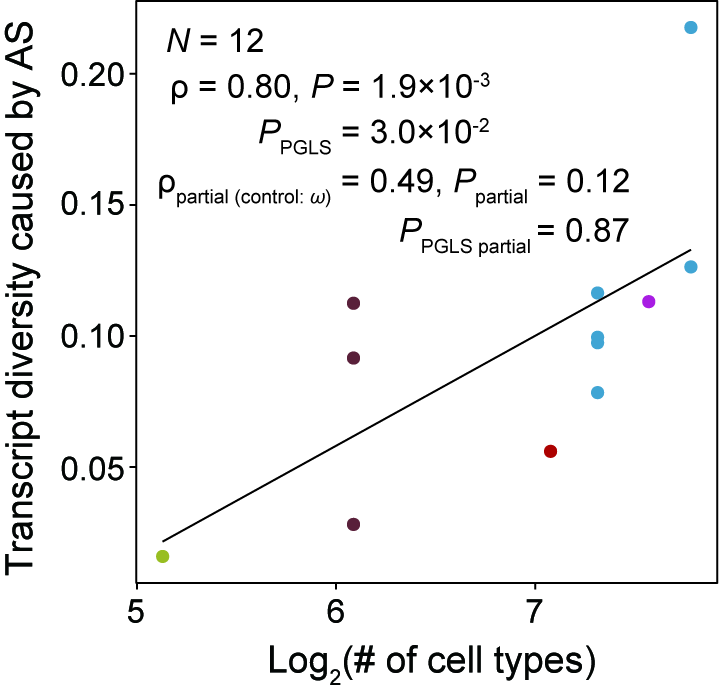

Supplement: S6 Fig — Dots represent the raw data. The data underlying this Figure can be found in https://doi.org/10.5281/zenodo.18514977. (TIF) [file pbio.3003671.s006.tif]
