# Supplementary material for: Transcript diversity reflects deleterious RNA processing errors shaped by population size in metazoans
Source: PLoS Biol. 2026 Mar 19;24(3):e3003671. doi: 10.1371/journal.pbio.3003671 (PMC13001929; doi:10.1371/journal.pbio.3003671)
Supplement: S2 Data — (DOCX) [file pbio.3003671.s008.docx]

**S2 Table.** The effective population size (*N*_e_) of 26 metazoans reported in previous studies.

| Species | *N*_e_ | Method | Reference |
| --- | --- | --- | --- |
| Anser brachyrhynchus | 6550 | θ/(2ν) | Ruokonen et al., 2005, Mol. Ecol.(1) |
| Apis mellifera | 366244 | θ/(3μ) | Wallberg et al., 2014, Nat Genet.(2) |
| Bombus terrestris | 2302940 | π/(4µ) | Lynch et al., 2017, Nature Rev. Genetics.(3) |
| Bos taurus | 90000 | π/(4µ) | MacEachern et al., 2009, BMC Genomics.(4) |
| Caenorhabditis briggsae | 267380 | π/(4µ) | Denver et al., 2012, Lynch et al., 2017.(3, 5) |
| Caenorhabditis elegans | 541379 | π/(4µ) | Denver et al., 2012, Lynch et al., 2017.(3, 5) |
| Canis lupus familiaris | 380000 | θ/(2μ) | Leonard et al., 2005, Molecular Ecology.(6) |
| Carlito syrichta | 15000 | π/(4µ) | Schmitz et al., 2016, Nat. Commun.(7) |
| Chlorocebus sabaeus | 12000 | π/(4µ) | Pfeifer SP., 2017, Evolution.(8) |
| Clupea harengus | 400000 | π/(4µ) | Feng et al., 2017, Elife.(9) |
| Daphnia pulex | 1175000 | π/(4µ) | Lynch et al., 2017, Nature Rev. Genetics.(3) |
| Drosophila melanogaster | 863020 | π/(4µ) | Lynch et al., 2017, Gossmann et al., 2012.(3, 10) |
| Drosophila pseudoobscura | 798607 | π/(4µ) | Gossmann et al., 2012, Genome Biol. Evol.(10) |
| Ficedula albicollis | 200000 | π/(4µ) | Smeds et al., 2016, Genome Res.(11) |
| Gallus gallus | 100000 | π/(4µ) | Axelsson et al., 2009, Mol Biol Evol.(12) |
| Gorilla gorilla | 35111 | π/(4µ) | Besenbacher et al., 2019, Scally et al., 2012.(13, 14) |
| Heliconius melpomene | 2068966 | π/(4µ) | Keightley et al., 2015, MBE.(15) |
| Homo sapiens | 24000 | π/(4µ) | Kong et al., 2012, Nature.(10, 16) |
| Macaca mulatta | 84336 | π/(4µ) | Xue et al., 2016, Genome Res.(17) |
| Microcebus murinus | 41000 | π/(4µ) | Campbell et al., 2019, Yoder et al., 2016.(18, 19) |
| Mus musculus | 177315 | π/(4µ) | Lynch et al., 2020, Charlesworth et al., 2009.(20, 21) |
| Ornithorhynchus anatinus | 41785 | π/(4µ) | Martin et al., 2018, MBE.(22) |
| Pan troglodytes | 28750 | π/(4µ) | Lynch et al., 2020, Charlesworth et al., 2009.(20, 21) |
| Pongo abelii | 56476 | π/(4µ) | Besenbacher et al., 2019, Nature Ecol. Evol.(13) |
| Pristionchus pacificus | 1750000 | π/(4µ) | Lynch et al., 2017, Nature Rev. Genetics.(3) |
| Rattus norvegicus | 130000 | π/(4µ) | Ness et al., 2012, G3.(23) |

**References**

1. M. Ruokonen, T. Aarvak, J. Madsen, Colonization history of the high-arctic pink-footed goose Anser brachyrhynchus. *Mol Ecol* **14**, 171-178 (2005).

2. A. Wallberg *et al.*, A worldwide survey of genome sequence variation provides insight into the evolutionary history of the honeybee Apis mellifera. *Nat Genet* **46**, 1081-1088 (2014).

3. M. Lynch *et al.*, Genetic drift, selection and the evolution of the mutation rate. *Nat Rev Genet* **17**, 704-714 (2016).

4. S. MacEachern, B. Hayes, J. McEwan, M. Goddard, An examination of positive selection and changing effective population size in Angus and Holstein cattle populations (Bos taurus) using a high density SNP genotyping platform and the contribution of ancient polymorphism to genomic diversity in Domestic cattle. *BMC Genomics* **10**, 181 (2009).

5. D. R. Denver *et al.*, Variation in base-substitution mutation in experimental and natural lineages of Caenorhabditis nematodes. *Genome Biol Evol* **4**, 513-522 (2012).

6. J. A. Leonard, C. Vila, R. K. Wayne, Legacy lost: genetic variability and population size of extirpated US grey wolves (Canis lupus). *Mol Ecol* **14**, 9-17 (2005).

7. J. Schmitz *et al.*, Genome sequence of the basal haplorrhine primate Tarsius syrichta reveals unusual insertions. *Nat Commun* **7**, 12997 (2016).

8. S. P. Pfeifer, Direct estimate of the spontaneous germ line mutation rate in African green monkeys. *Evolution* **71**, 2858-2870 (2017).

9. C. Feng *et al.*, Moderate nucleotide diversity in the Atlantic herring is associated with a low mutation rate. *Elife* **6** (2017).

10. T. I. Gossmann, P. D. Keightley, A. Eyre-Walker, The effect of variation in the effective population size on the rate of adaptive molecular evolution in eukaryotes. *Genome Biol Evol* **4**, 658-667 (2012).

11. L. Smeds, A. Qvarnstrom, H. Ellegren, Direct estimate of the rate of germline mutation in a bird. *Genome Res* **26**, 1211-1218 (2016).

12. E. Axelsson, H. Ellegren, Quantification of adaptive evolution of genes expressed in avian brain and the population size effect on the efficacy of selection. *Mol Biol Evol* **26**, 1073-1079 (2009).

13. S. Besenbacher, C. Hvilsom, T. Marques-Bonet, T. Mailund, M. H. Schierup, Direct estimation of mutations in great apes reconciles phylogenetic dating. *Nat Ecol Evol* **3**, 286-292 (2019).

14. A. Scally *et al.*, Insights into hominid evolution from the gorilla genome sequence. *Nature* **483**, 169-175 (2012).

15. P. D. Keightley *et al.*, Estimation of the spontaneous mutation rate in Heliconius melpomene. *Mol Biol Evol* **32**, 239-243 (2015).

16. A. Kong *et al.*, Rate of de novo mutations and the importance of father's age to disease risk. *Nature* **488**, 471-475 (2012).

17. C. Xue *et al.*, The population genomics of rhesus macaques (Macaca mulatta) based on whole-genome sequences. *Genome Res* **26**, 1651-1662 (2016).

18. C. R. Campbell *et al.*, Pedigree-based and phylogenetic methods support surprising patterns of mutation rate and spectrum in the gray mouse lemur. *Heredity (Edinb)* **127**, 233-244 (2021).

19. A. D. Yoder *et al.*, Geogenetic patterns in mouse lemurs (genus Microcebus) reveal the ghosts of Madagascar's forests past. *Proc Natl Acad Sci U S A* **113**, 8049-8056 (2016).

20. M. Lynch, B. Trickovic, A Theoretical Framework for Evolutionary Cell Biology. *J Mol Biol* **432**, 1861-1879 (2020).

21. B. Charlesworth, Fundamental concepts in genetics: effective population size and patterns of molecular evolution and variation. *Nat Rev Genet* **10**, 195-205 (2009).

22. H. C. Martin *et al.*, Insights into Platypus Population Structure and History from Whole-Genome Sequencing. *Mol Biol Evol* **35**, 1238-1252 (2018).

23. R. W. Ness *et al.*, Nuclear gene variation in wild brown rats. *G3 (Bethesda)* **2**, 1661-1664 (2012).
